# Insights into pediatric lupus nephritis: clinical features and short-term outcomes from a single center retrospective study

**DOI:** 10.1186/s12882-025-04059-6

**Published:** 2025-03-22

**Authors:** Sabeeta Khatri, Irshad Ali Bajeer, Madiha Aziz, Mohammed Mubarak, Ali Asghar Lanewala, Seema Hashmi

**Affiliations:** Sindh Institute of Urology and Transplantation, Karachi, Pakistan

**Keywords:** Lupus nephritis, Pediatric, Outcome and immunosuppression

## Abstract

**Background:**

Pediatric lupus nephritis is a rare glomerular disease with paucity of data on short and long term outcomes. This single center study aims to assess the outcomes at 12 months and the last follow-up visit.

**Methods:**

This retrospective review of medical charts was done to include children diagnosed with lupus nephritis at Sindh Institute of Urology and Transplantation Karachi from July, 2015 to December, 2022.

**Results:**

Twenty five children included in the analysis had mean age of 11.5 ± 3.5 years with predominant 20 (80%) girls. The most common clinical presentation was nephrotic syndrome in 15 (60%). The means of estimated GFR and serum albumin improved from baseline to 12 months, however serum albumin showed statistically significant improvement (121 ml/min/1.73 m^2^ ± 77 to 130 ml/min/1.73 m^2^ ± 57, –9.2, p-value 0.53 and 2.1 gm/dl ± 0.81 to 3.5 ± 0.73, − 1.4 p-value 0.00). The choice of induction drug had no impact on composite outcome with similar complete remission rates in MMF versus Cyclophosphamide and Calcineurin inhibitors groups (4/10, 40% versus 6/15,40%; p-value 0.81). The failure of complete remission of proteinuria at 12 months was statistically associated with poor composite outcome at last follow-up visit (p-value 0.02).

**Conclusion:**

In our study, the choice of induction regimens had no impact on overall outcome. However, we identified the importance of targeting and reducing proteinuria to improve outcomes in pediatric patients with lupus nephritis.

**Supplementary Information:**

The online version contains supplementary material available at 10.1186/s12882-025-04059-6.

## Background

Systemic lupus erythematosus (SLE) is a chronic, autoimmune, inflammatory disease with a relapsing-remitting course and multisystem involvement. The pediatric-onset SLE (pSLE) represents 10–20% of all SLE cases, with an annual incidence of 0.3–0.9 per 100,000 children-years, and prevalence rate of 3.3–8.8 per 100,000 children [1, 2]. Lupus nephritis (LN) in pSLE is more common than adults and is seen in nearly 50% of pSLE patients with variable presentation as asymptomatic urinary abnormalities, acute kidney injury and chronic kidney disease [3]. Children less than 18 year experience higher morbidity and mortality with lupus nephritis [4]. The percentage of End stage kidney disease (ESKD) patients in lupus nephritis is up to 10% and patients with renal involvement have higher mortality as compared to patients with non-renal involvement [5].

The treatment consists of two phases induction and maintenance. Since 1980s, numerous treatment trials have been conducted. Among them, the national institute of health is a landmark study. It involved comparing the effectiveness of monthly intravenous cyclophosphamide (CYC) plus prednisone (PRED) with azathioprine (AZA) plus PRED or PRED alone. The results revealed that treatment with CYC led to fewer instances of relapse and reduced risk of ESKD. Nonetheless, concerns arose regarding treatment related complications such as infection, ovarian failure, hemorrhagic cystitis, bladder cancer, mortality associated with the treatment [6]. The need for treatment with less toxic and equally effective therapy had motivated the researchers to study new medications and distinct methods of CYC administration [7].

In the Euro–lupus trial, the efficacy of less intensive regimen has been evaluated. This trial compared shorter (lower dose) and longer (higher dose) intravenous CYC therapy in white patients and found that the low-dose CYC regimen was comparable in efficacy to a higher dose regimen in patients with less severe proliferative lupus nephritis [8]. The Aspreva Lupus Management Study (ALMS) compared induction therapy with CYC or Mycophenolate mofetil (MMF) and concluded that MMF is as effective as CYC [9]. However, ALMS failed to meet its primary end point of superiority of MMF. In a meta-analysis that included 45 trials, MMF had comparable efficacy to CYC for induction phase in proliferative lupus nephritis [10]. There are few studies comparing the drugs for maintenance therapy. Short and long-term follow-up of MAINTAIN trail and extension phase of ALMS trial do not favor one single agent. Therefore, AZA and MMF are equally effective. However, real world scenario has a greater preference for MMF [11–13]. According to the Kidney Disease Improving Global outcome (KDIGO) clinical practice guidelines, American College of Rheumatology (ACR), and the joint European League Against Rheumatism and European Renal Association- European Dialysis and transplant association (EULAR/ERA-EDTA) guidelines initial treatment should consist of glucocorticoids along with either CYC or MMF [14–16].

There are limited studies available on outcome of childhood lupus nephritis in developing countries [17, 18]. The consensus treatment plan by Childhood Arthritis and Rheumatology Research Alliance (CARRA) and Single Hub and Access point for Pediatric Rheumatology in Europe (SHARE) are based on single center retrospective studies and adult literature [19, 20]. Taking into account this knowledge deficit, the primary objective of this study is to determine the outcome of pediatric lupus nephritis at 12 months and last follow-up visit.

## Materials and methods

A retrospective cohort study was carried out at the Pediatric Nephrology department, Sindh Institute of Urology and Transplantation (SIUT) Karachi from July, 2015 to December, 2022. The project was approved by the ethical review committee. Children less than 18 years diagnosed as biopsy proven LN class III, IV, V, III + V and IV + V at SIUT were included. Children with the duration of follow-up less than one year and full house nephropathy (Negative ANA) were excluded.

A review of medical charts was done to include all the demographic and clinical profile. Laboratory parameters like renal functions, serum albumin, urinary studies and immunological tests including ANA, double stranded DNA and extractable nuclear antigen were documented. Estimated glomerular filtration rate (eGFR) was calculated at each visit through Schwartz formula. Serum creatinine is measured through Jaffe method so appropriate constants were used [21]. Renal biopsies at our center were carried out under real time ultrasound guidance using 18 or 16 gauge needles. Samples were stained with basic stains for immunofluorescence. Biopsies were classified as per ISN/RPS classification and NIH activity and chronicity scores was calculated [22, 23].

The induction phase included Methylprednisolone 3 pulses followed by daily steroids for 6 to 8 weeks and intravenous Cyclophosphamide 500 mg/m^2^ monthly for 3 to 6 months. Alternatively, we also prescribed MMF and calcineurin inhibitors in the induction phase. The maintenance phase included one of AZA, MMF or calcineurin inhibitors (CNI) along with low dose PRED. Additionally, all the children received hydroxychloroquine and Enalapril. Children declared non responder to the initial immunosuppressant at 3–6 months were switched to alternate drug (Cyclophosphamide to MMF or vice versa). Repeat kidney biopsies were carried out if no response was documented despite adequate immunosuppression for 6 to 9 months. Laboratory parameters like complete blood count, renal functions, serum albumin and urinary studies were monitored every 4–12 weekly.

The diagnosis of LN was based on nephrotic or nephritic feature, positive ANA with either positivity of dsDNA or ENA and renal biopsy findings consistent with LN. The primary outcome was measured at the 12 month and last follow-up visit. The definition of each of the outcome parameter is pediatric population is not established. The definition we used in our cohort is shown in Table 1.

**Table 1:** Definitions of outcome measures

Glomerular Filtration Rate	Complete: GFR > 90 ml/min/1.73 m^2^ and not less than 20% below baseline
	Partial: At least 50% improvement in the GFR but < 90 ml/min/1.73 m^2^
	No response: not fulfilling above criteria for complete or partial response
Proteinuria	Complete: Negative or trace proteinuria on urinary dipstick
	Partial: Any degree of proteinuria on dipstick with serum albumin > 3 gm/dl
	No response: not fulfilling above criteria for complete or partial response
Composite outcome	Complete: Both GFR and Proteinuria criteria in the complete response category
	Partial: Complete or partial response to GFR along with partial response to proteinuria
	No response: not fulfilling above criteria for complete or partial response

GFR, Glomerular filtration rate; gm, gram

The flare of LN was defined as worsening or recurrence of proteinuria, hematuria, and drop in serum albumin level or rise in serum creatinine. The assessments at 3, 6, 12, 24, 36, 48 and last follow-up included blood pressure, GFR, albumin and urine dipstick for proteinuria.

All descriptive data was entered into SPSS version 26. The quantitative variables with normal distribution were expressed as mean with standard deviation (± SD) and rest as median with interquartile range (IQR). Qualitative variables were expressed as percentages or range. Chi-square was used for comparison of categorical variables. P-value < 0.05 was considered as significant.

## Results

A total of 25 children had complete demographic, clinical, kidney biopsy and 12 months follow-up data for the primary outcome as shown in Table 2.

**Table 2:** Baseline demographics of study participants

Parameter	Value
Age (Years)	11.5 (± 3.46)
Sex (girls)	20 (80%)
BMI (Kg/m^2^)	15.5 (13.9–17.5)
Baseline eGFR (ml/min/ 1.73 m^2^)	121 (± 77)
Baseline serum Albumin (gm/dl)	2.1 (± 0.81)
Extra renal involvement	
Skin and Mucosa	9 (36%)
Arthritis	5 (20%)
CNS	4 (16%)
Anemia and Thrombocytopenia	4 (16%)
Duration of follow-up (months)	45.52 ± 21.43

BMI, Body mass index; CNS, central nervous system

Mean age of the participants was 11.5 ± 3.4 years with predominant 20 (80%) girls. In the initial visit 10 (40%) were hypertensive. The most common presentation was with nephrotic syndrome in 15 (60%), out of them three children were biopsied due to steroid resistant nephrotic syndrome. Acute glomerulonephritis was documented in 9 (36%) children. A 12 years girl (4%) was admitted with clinical features of IgA vasculitis (formerly Henoch-Schonlein Nephritis) with bilateral lower limb purpuric rash and arthritis. Her immunological workup and kidney histology were consistent with LN class IV.

The mean eGFR at baseline was 121 ml/min/1.73 m^2^ ± 77 and it slightly improved to 130 ml/min/1.73 m^2^ ± 57 at 12 months. However, the mean difference was not statistically significant (–9.2, p-value 0.53). Serum albumin had shown statistically significant difference in the means at baseline and 12 months (2.1 gm/dl ± 0.81 versus 3.5 ± 0.73, − 1.4 p- value 0.00). Complements were tested in all the children and 14 (56%) had both C3 and C4 below normal range. In the remaining children, 7 (28%) had low C3, 1 (4%) low C4 and 3 (12%) showed normal levels of both. All the children tested positive for ANA, dsDNA was positive in 21 (84%). Four (17%) children were investigated for extractable nuclear antigen which turned out to be positive. Antiphospholipid antibody (APLA) was positive in 4 (31%) out of 13 tested in the cohort.

In the histopathology the most common class of lupus nephritis was IV in 9 (36%), followed by combined IV and V in 7 (28%) and another 6 (24%) had class III and V. Two (8%) were categorized as class V and one (4%) had class III. The NIH activity and chronicity scores were calculated which were reported as median 2 (IQR 0.5–4) and 1 (IQR 1–2) respectively. The immunofluorescence studies were carried out on fresh tissue and full house pattern was seen in 14 (56%) and rest had non-full house immune-complex in 9 (36%) and 2 (8%) had pauci-immune pattern. All 9 children with non-full house immune-complex were positive for ANA and 8 out of 9 had positive dsDNA and 1 out of 9 anti-smith positive. Based on these findings, the possibility of alternate diagnoses in these children is minimal. Two children with pauci-immune histopathology had positive ANA, dsDNA and both C3 and C4 were low.Interestingly, a 12 years boy with pauci-immune pattern had demonstrated positivity of MPO ANCA. On subsequent visits it tested negative.

In the induction phase all the children received methylprednisolone along with pulse CYC in 13 (52%) and MMF in 10 (40%). Two children (8%) both with class V were induced and maintained with CNI. Majority of the children 20 (80%) were prescribed MMF as maintenance therapy and 3 (12%) Azathioprine. All the children took hydroxychloroquine and Enalapril therapy. No additional therapy was considered in APLA positive children due to the absence of thromboembolic event. The child with MPO ANCA positivity had presented after the publication of PEXIVAS trial so standard immunosuppression was considered without plasma exchange [24].

**Fig. 1:**
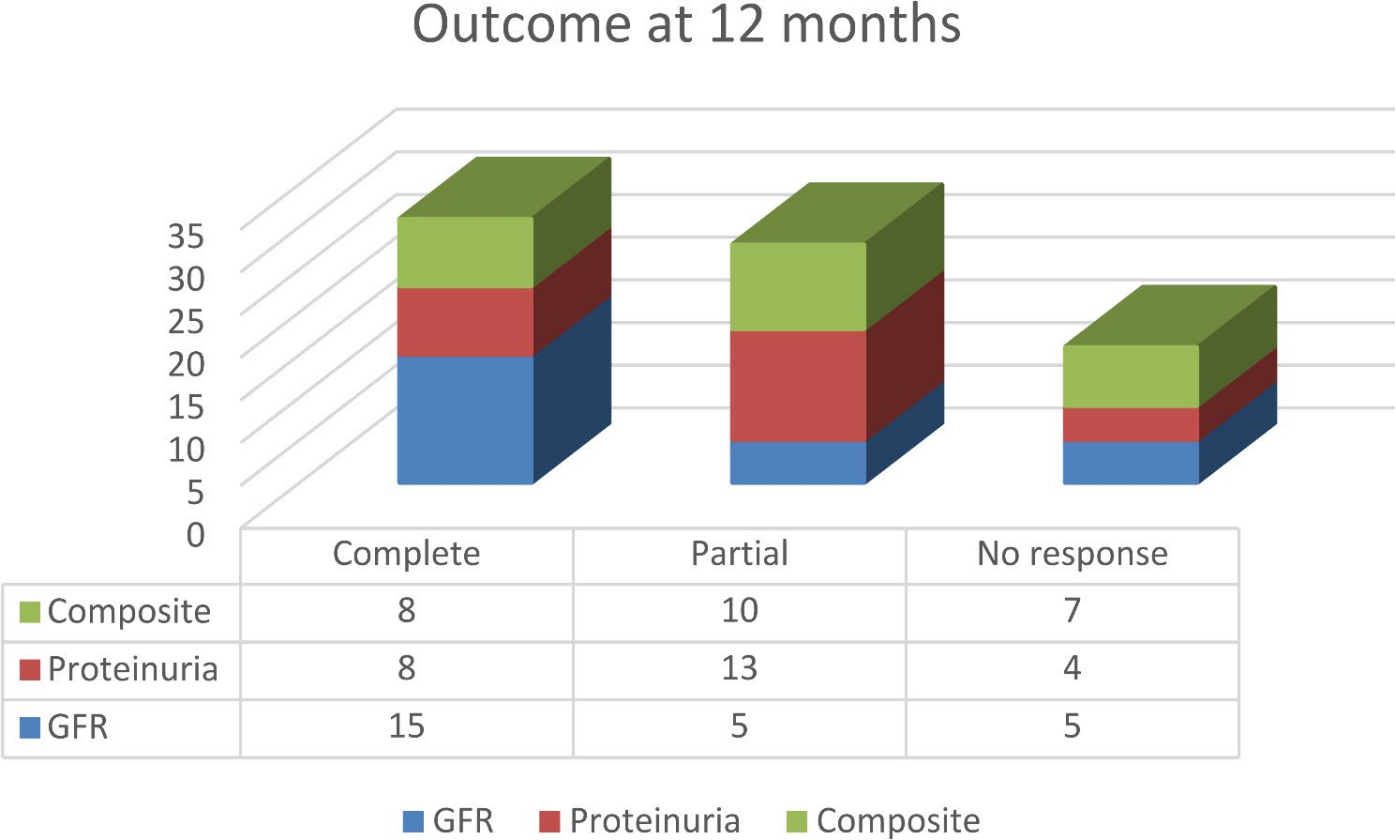
Outcome of lupus nephritis at 12 months

**Fig. 2:**
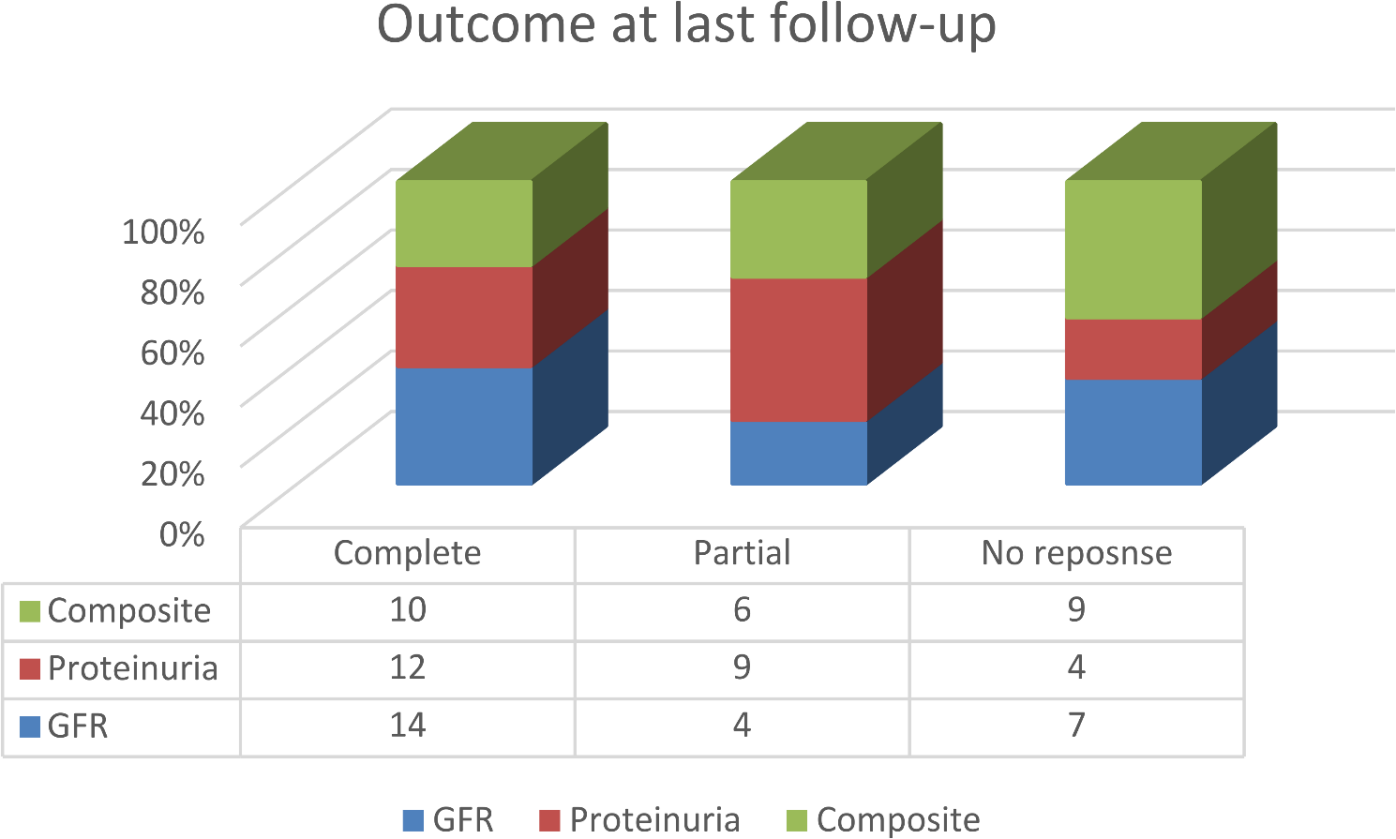
Outcome of lupus nephritis at last follow-up visit

The primary outcome of the treatment was assessed at 12 months and last available follow-up visit in terms of complete, partial or no response to GFR improvement, reduction in proteinuria and composite outcome of both Figs. 1 and 2.

The entire cohort of 25 children, diagnosed between 2015 and 2022 had cumulative follow-up of 1118 months with mean of 45.5 ± 21.4 per child. The renal flare of the disease was relatively infrequent with one flare per 111 months of follow-up. Four children (three with class IV and one class III and V) required repeat biopsy due to non-response despite adequate immunosuppression for 6–9 months. Out of 4 children, two each had received CYC and MMF as the induction drugs. The biopsy reports demonstrated no change in all three with class IV. While one with class III and V had switched to IV and V. One of the 4 children had extrarenal features of oral ulcer, arthritis and hematological manifestation. A boy (4%) had progressed to end stage kidney disease requiring maintenance hemodialysis. He had presented with GFR of 43 ml/min/1.73 m^2^, lupus nephritis class IV with chronicity index of 7. His induction therapy included pulse methylprednisolone and Cyclophosphamide, followed by MMF and per oral prednisolone. Considering the chronicity index escalation of immunosuppression was withheld. The choice of induction drug had no impact on composite outcome with similar complete remission rates in MMF versus CYP/CNI groups (4/10, 40% versus 6/15,40%; p-value 0.81). The outcome with respect to class of lupus nephritis is described in the Table 3.

**Table 3:** The outcome with respect to class of lupus nephritis

Outcome at 12 months					
	Class III (*n* = 1)	Class III + V (*n* = 6)	Class IV (*n* = 9)	Class IV + V (*n* = 7)	Class V (*n* = 2)
CR	1	2	4	2	1
PR	0	1	2	3	1
NR	0	3	1	1	0
ESKD	0	0	1	0	0
Death	0	0	1	1	0
Outcome at last follow-up visit					
CR	1	1	4	3	1
PR	0	3	1	1	1
NR	0	2	2	2	0
ESKD	0	0	1	0	0
Death	0	0	1	1	0

CR, complete remission; PR partial remission; NR, no response; ESKD, end stage kidney disease

Composite no response at last follow-up was common in children presenting with rapidly progressive glomerulonephritis 5 (56%) than with nephrotic syndrome 3 (20%). Seven children (28%) were in complete remission of proteinuria at 12 months. Of the remaining 17 (72%), 3 (18%) were able to achieve composite complete response at the last visit. So, the failure of complete remission of proteinuria at 12 months was statistically associated with poor composite outcome at last follow-up visit (p-value 0.02). The flare of disease had poor composite complete response rate at 12 months with 4 out of 10 (40%) with disease flare had complete response (p-value 0.005).

Out of 25, 5(20%) had reversible leukopenia and 3 (12%) developed sepsis/septic shock. Two participants (8%) had mortality in the study duration, one each due to septic shock and dengue hemorrhagic fever. The common characteristics of individual patients have been described as a Table 1 in the supplementary section.

## Discussion

This study analyzed the clinicopathologic features and short-term outcome of pediatric biopsy-proven lupus nephritis patients registered over a period of 7 years and followed up for at least 12 months. Two-thirds of our patient remained in remission at the last available follow-up. The choice of treatment regime had no effect on the outcome, however, renal failure at presentation portended a poor outcome.

The demographic features in this cohort were comparable to other Asian studies. The mean age was 11.5 ± 3.46 years which was similar to that reported from multiple studies across the globe [25]. A slightly higher mean age of 13 years was reported in the Indian cohort by Srivastava R et al. and the Chinese group by Chan EY et al. [26]. Lupus primarily affects girls and 80% of our cohort was female, this can range from 60 to 80% as reported by other studies [27].

Half of the patients presented with renal impairment while one-third of them came to attention due to extra-renal lupus manifestations and were found to have renal involvement on work-up. Chan Y et al. reported similar findings in their report albeit with a slight difference [26]. 27% of their cohort presented with nephrotic syndrome while a lesser percentage of 16% presented with steroid resistant nephrotic syndrome in our cohort. This difference could perhaps be explained by ethnic differences.

Hypocomplementemia was seen in most patients with low C3 in 84% of them, while 60% of them also had low C4. Between the two, low C3 strongly associates with nephritis and renal damage as reported by Durcan and colleagues [28]. Normal C4 levels and absence of anti dsDNA antibodies, as seen in a small percentage of our patients, has been observed in other cohorts too. It signifies an absence of classical pathway activation; instead, it has been postulated that alternate nonimmune complex-dependent complement pathway activation also occurs in a significant population of lupus patients [29].

Class IV lupus nephritis was the commonest histopathological pattern reported in our cohort as has been reported by almost all studies on lupus nephritis [25–27]. In contrast, only 21% of the renal biopsies in the 2 decade long Chinese study were reported to have proliferative mixed class; while more than half of the biopsies at 52% were reported as mixed class in our cohort. This could perhaps be explained by greater renal involvement as 50% of our patients presented with some degree of renal impairment. Besides, the median activity and chronicity score were lower in our cohort but a higher median activity score of ‘7’ was reported by Hari P et al. and colleagues in their Indian cohort [27].

While full house pattern of immunofluorescence is predominant in lupus nephritis, pauci-immune and ANCA positive lupus nephritis respectively have been reported in 15-20% of cases with variable outcomes [30, 31]. Rarely, both have been reported together too [32]. Our cohort had 2(8%) patients with pauci-immune histology and one of them was ANCA positive. Both patients achieved remission.

Definitions used for renal remission are variable across studies. Various studies have reported between 50% and 78.8% of the children to be in complete remission depending on the definition used [33]. In concurrence, 72% of our patients were in complete or partial remission at 12 months follow-up as has been documented by Chan et al. in Chinese children and similarly by Silva et al. in Portugese children [34].

These definitions are by no means complete and further long term studies are required to classify and define disease progression and remission parameters. Besides, choice of treatment regimen has not affected outcomes.

Mortality rates and risk of end stage kidney disease have improved worldwide over the past half a century with the use of immunosuppression in lupus nephritis. Mortality rates of 6- 21% have been reported from various global centers [33]. Our center had a mortality of 8% with infection being the major cause of death as seen in other centers too. We reported 4% children progressing to end stage kidney disease while up to 15% progression to ESKD has been reported in other studies [35, 36].

In conclusion, while the outcomes of children with lupus nephritis have improved after use of immunosuppression, long term studies are still required to improve understanding and treatment goals.

## Electronic supplementary material

Below is the link to the electronic supplementary material.

Supplementary Material 1

## Data Availability

Data will be provided upon the request.
