# Supplementary material for: Insights into pediatric lupus nephritis: clinical features and short-term outcomes from a single center retrospective study
Source: BMC Nephrol. 2025 Mar 22;26:145. doi: 10.1186/s12882-025-04059-6 (PMC11929978; doi:10.1186/s12882-025-04059-6)
Supplement: Supplementary file 1 — Supplementary Material 1 [file 12882_2025_4059_MOESM1_ESM.docx]

Table 1. Description of all the included study participants

| Patient Number | Age | Sex | Presenting feature | Class | Induction drug | Maintenance  drug | Outcome at 12 months | Outcome at last follow-up |
| --- | --- | --- | --- | --- | --- | --- | --- | --- |
| 1 | 10 | Female | Nephritic Syndrome | IV + V | MMF | MMF | CR | CR |
| 2 | 14 | Female | Nephritic Syndrome | IV | CYC | MMF | CR | PR |
| 3 | 10 | Male | Nephritic Syndrome | IV | CYC | MMF | PR | NR |
| 4 | 12 | Male | Nephritic Syndrome | IV | CYC | MMF | ESKD | ESKD |
| 5 | 16 | Female | Nephrotic Syndrome | III | CYC | AZA | CR | CR |
| 6 | 11 | Female | Nephritic Syndrome | IV + V | CYC | MMF | PR | NR |
| 7 | 10 | Female | Nephrotic Syndrome | IV | MMF | MMF | NR | NR |
| 8 | 14 | Female | Nephritic Syndrome | IV + V | MMF | MMF | PR | PR |
| 9 | 18 | Male | Nephritic Syndrome | IV + V | CYC | MMF | NR | NR |
| 10 | 12 | Female | HSP | IV | CYC | MMF | Expired | Expired |
| 11 | 17 | Female | Nephrotic Syndrome | IV | CYC | MMF | CR | CR |
| 12 | 12 | Female | Nephrotic Syndrome | IV + V | MMF | MMF | PR | CR |
| 13 | 13 | Male | Nephritic Syndrome | IV | CYC | AZA | CR | CR |
| 14 | 16 | Female | Nephrotic Syndrome | IV | MMF | MMF | PR | CR |
| 15 | 12 | Female | Nephrotic Syndrome | V | CNI | CNI | PR | CR |
| 16 | 4 | Female | Nephritic Syndrome | III + V | MMF | MMF | NR | NR |
| 17 | 9 | Female | Nephrotic Syndrome | III + V | MMF | MMF | PR | PR |
| 18 | 10 | Female | Nephrotic Syndrome | III + V | CYC | MMF | CR | CR |
| 19 | 14 | Female | Nephrotic Syndrome | IV + V | MMF | MMF | CR | CR |
| 20 | 8 | Female | Nephrotic Syndrome | III + V | MMF | MMF | NR | NR |
| 21 | 11 | Female | Nephrotic Syndrome | III + V | MMF | MMF | NR | PR |
| 22 | 8 | Female | Nephrotic Syndrome | IV + V | CYC | AZA | Expired | Expired |
| 23 | 12 | Male | Nephrotic Syndrome | IV | CYC | MMF | CR | CR |
| 24 | 12 | Female | Nephrotic Syndrome | III + V | CYC | MMF | CR | PR |
| 25 | 4 | Female | Nephrotic Syndrome | V | CNI | CNI | CR | PR |

^CYC, Cyclophosphamide; MMF, Mycophenolate Mofetil; CNI, Calcineurin inhibitors; AZA, Azathioprine; CR, Complete remission; PR, Partial remission; NR, Non-responder; ESKD, End stage kidney disease; HSP, Henoch-Schonlein purpura^
